# Short-term clinical and manometric outcomes of percutaneous tibial nerve stimulation for faecal incontinence: a large single-centre series

**DOI:** 10.1007/s10151-024-02916-4

**Published:** 2024-04-03

**Authors:** A. O’Connor, C. Molyneux, K. Foster, G. Faulkner, A. Sharma, E. Kiff, D. H. Vasant, K. Telford

**Affiliations:** 1 Department of Colorectal Surgery, Manchester University NHS Foundation Trust, Wythenshawe Hospital, 2nd Floor Acute Block, Southmoor Road, Manchester, M23 9LT UK; 2 Faculty of Biology, Medicine, and Health, The University of Manchester, Manchester, UK; 3 Neurogastroenterology Unit, Gastroenterology, Wythenshawe Hospital, Manchester University NHS Foundation Trust, Manchester, UK; 4 Division of Diabetes, Endocrinology and Gastroenterology, The University of Manchester, Oxford Road, Manchester, UK

**Keywords:** Faecal incontinence, Percutaneous tibial nerve stimulation, High resolution anorectal manometry, Quality of life

## Abstract

**Background:**

Faecal incontinence (FI) is common, with a significant impact on quality of life. Percutaneous tibial nerve stimulation (PTNS) is a therapy for FI; however, its role has recently been questioned. Here we report the short-term clinical and manometric outcomes in a large tertiary centre.

**Methods:**

A retrospective review of a prospective PTNS database was performed, extracting patient-reported FI outcome measures including bowel diary, the St Marks’s Incontinence Score (SMIS) and Manchester Health Questionnaire (MHQ). Successful treatment was > 50% improvement in symptoms, whilst a partial response was 25–50% improvement. High-resolution anorectal manometry (HRAM) results before and after PTNS were recorded.

**Results:**

Data were available from 135 patients [119 (88%) females; median age: 60 years (range: 27–82years)]. Overall, patients reported a reduction in urge FI (2.5–1) and passive FI episodes (2–1.5; *p* < 0.05) alongside a reduction in SMIS (16.5–14) and MHQ (517.5–460.0; *p* < 0.001). Some 76 (56%) patients reported success, whilst a further 20 (15%) reported a partial response. There were statistically significant reductions in rectal balloon thresholds and an increase in incremental squeeze pressure; however, these changes were independent of treatment success.

**Conclusion:**

Patients report PTNS improves FI symptoms in the short term. Despite this improvement, changes in HRAM parameters were independent of this success. HRAM may be unable to measure the clinical effect of PTNS, or there remains the possibility of a placebo effect. Further work is required to define the role of PTNS in the treatment of FI.

## Introduction

Faecal incontinence (FI) is a common condition negatively affecting an individual’s physical, emotional and social wellbeing alongside posing a significant public health burden [1, 2]. Its incidence increases with advancing age with a prevalence of 2–21% in the community, and it remains a common cause of admission to a residential care facility [3].

In the management of FI that fails to respond to conservative interventions, neuromodulation therapies have evolved as treatment options for FI that are safe, reversible and minimally invasive [4]. Sacral neuromodulation (SNM) is now the first-line surgical intervention for FI with demonstrable short and long-term efficacy [4, 5]; however, it requires two surgical procedures, and carries a high upfront financial cost. By contrast, percutaneous tibial nerve stimulation (PTNS) is an outpatient, minimally invasive therapy involving stimulation of the tibial nerve at the ankle [6], which may be used as a treatment option before SNM [7]. Given the shared sacral nerve root origin of the pudendal nerve and posterior tibial nerve, it is believed stimulation of the latter could lead to the improvement in FI symptoms reported with SNM [8].

The reported short-term clinical success of PTNS ranges from 62% to 83% using various outcome measures, including bowel diaries and questionnaires [6]. However, the efficacy of PTNS has been challenged by the Control of Faecal Incontinence using Distal Neuromodulation (CONFIDeNT) trial [9] suggesting PTNS did not have significant benefit over sham electrical stimulation. These results were later questioned as, with the exclusion of patients with obstructive defecation, a significant clinical effect of PTNS was seen [10]. Indeed, PTNS remains included in treatment pathways for FI management as a procedure of low risk with possible clinical benefit [11, 12].

The aim of this study was to establish the short-term clinical outcomes of PTNS in a large single-centre tertiary referral unit, and investigate the physiological changes measured with high resolution anorectal manometry (HRAM).

## Materials and methods

A retrospective review of a prospectively managed neuromodulation database was performed at a tertiary referral pelvic floor unit to identify patients treated with PTNS for FI. Patients with FI who were offered PTNS but who declined are not recorded in this database. All patients were reviewed by a colorectal surgeon who obtained a full history and performed a clinical examination. Symptoms of faecal urgency, faecal incontinence (urge and passive) and evacuatory difficulties were recorded. Evacuatory difficulties included any symptom of patient-perceived difficulty in evacuation, regular digitation, a sensation of incomplete emptying or excessive straining. Patients had already received relevant conservative interventions, including lifestyle adjustments, pharmacological therapy and biofeedback treatment. Patients were routinely discussed at the pelvic floor multidisciplinary team meeting before PTNS therapy was offered. Data were extracted from the database including patient demographics, baseline clinical data, symptomatology, relevant investigation results, the number of PTNS treatments, FI outcome measures and HRAM results before and after treatment. Patients were excluded from the analysis if at least one of the same outcome measures recorded before and after treatment were not available, making it impossible to assess treatment efficacy.

### Percutaneous tibial nerve stimulation

In our unit, PTNS is delivered in 12 weekly consecutive 30-min treatments with the Urgent PC^®^ Neuromodulation system (Laborie^®^, NH, USA). Patients are seated with a leg elevated. The electrode needle is placed percutaneously 2 cm deep to the skin, 5 cm cephalad to the medial malleolus and 2 cm posterior to the tibia. A surface electrode is placed on the ipsilateral foot, medial to the calcaneum. Correct placement is confirmed by demonstrating either a motor response (plantar flexion of the great toe) or a sensory response (tingling to the toes, arch or heel) through incremental stimulation increases[9, 13].

### High-resolution anorectal manometry

HRAM was performed using a water-perfused system incorporating 10 circumferential pressor sensors at 0.8 cm intervals with an external diameter of 14 Fr (Mui Scientific™, ON, Canada). Prior to the investigation, the pressure was zeroed to atmospheric pressure at the anal verge. The investigation was performed with the patient in the left lateral position. A digital rectal examination was performed, and patients asked to ‘squeeze’ and ‘push’ to confirm their understanding of these instructions before HRAM was performed according to the standardised London protocol [14]. Mean resting pressure (MRP), maximum squeeze pressure (MSP) and incremental squeeze pressure (ISP) were recorded in mmHg. Rectal sensory volumes were recorded during balloon inflation as patients indicated their first sensation (onset), desire to defecate/call to stool (call) and maximum tolerated volume (urge) in ml. HRAM is commonly performed before starting PTNS as part of the routine tests for the investigation of FI. Since PTNS was introduced in our unit, HRAM has been repeated between 6–12 weeks following completion of the treatment.

### Outcome assessment

Treatment outcomes were assessed using a 2-week bowel diary to calculate weekly faecal urgency episodes (FUE), urge faecal incontinence episodes (UFIE) and passive faecal incontinence episodes (PFIE) alongside the St Mark’s Incontinence Score (SMIS), and the Manchester Health Questionnaire (MHQ). The SMIS is an FI-specific symptom severity questionnaire with scores ranging from ‘0’ representing perfect continence to ‘24’ representing complete incontinence [15]. The MHQ is a validated FI-specific questionnaire measuring quality of life (QoL) impact across nine domains: overall health, overall impact of FI on life, physical limitations, social limitations, relationship impact, emotional impact, sleep and energy impact, and overall FI severity [16]. Scores range from ‘0’ representing no impact on QoL to a maximum score of ‘900’. Patients are routinely asked to complete all three outcome measures before starting PTNS and between 6 and 12 weeks after PTNS treatment to assess the response. Outcome measures are completed at home without healthcare professional supervision; however, if unclear responses are provided, the result is confirmed with the patient at the next available clinic appointment. Patients were stratified into three groups according to the percentage improvement with any one of these outcome measures:

-  < 25% (failure)
- 25–50% (partial response)
-  > 50% (success)

### Statistical analysis

Statistical analysis was performed using SPSS^®^ for Mac^®^ (version 29.0, IBM^®^, NY, USA). Data are presented as median (interquartile range [1st quartile–3rd quartile]) unless indicated. The Wilcoxon signed-rank sum test was used for comparisons of paired data and the Kruskal–Wallis test for comparisons of unpaired data. Chi-squared was used to examine the distribution of categorical variables between groups. Statistical significance considered at the *p* < 0.05 level.

## Results

The neuromodulation database was searched in April 2023 to identify patients treated with PTNS for FI. A total of 158 patients were identified as having completed PTNS from September 2013 to February 2023. Excluding 23 (15%) patients with incomplete outcome measures, data from 135 patients (119 [88%] females, median age: 60 [range: 27–82]) were available for analysis. Of these, 127 completed all 12 PTNS sessions, with the remaining patients completing either 11 sessions (*n* = 7) or 9 sessions (*n* = 1). Baseline demographic and clinical data are presented in Table 1. Most of the female patients were parous (110/119, 92%) with a history of at least one vaginal delivery (108/110, 98%). The predominant symptom among all patients was faecal urgency (126/135, 93%). Four patients had been previously treated with SNM and subsequently underwent PTNS either due to poor SNM efficacy (*n* = 2) or whilst awaiting SNM revision surgery (*n* = 2). Three further patients had previously undergone percutaneous nerve evaluation, with two reporting a successful trial but choosing PTNS treatment in favour of permanent SNM implantation. A total of 60 patients were investigated with defecating proctography and 112 underwent endoanal ultrasound prior to PTNS as part of their routine investigations for FI (Table 1).

**Table 1:** Baseline patient characteristics

Variable	Number of patients (*N* = 135)
Age, median (range)	60 (27–82)
Male/female, *n* (%)	16 (12%)/119 (88%)
Obstetric history, *n* (%)	
Parous*	110 (92%)
Vaginal delivery^	108 (98%)
Caesarean section^	11 (10%)
Episiotomies or perineal tear^+^	82 (76%)
Bowel function history, *n* (%)	
Faecal urgency	126 (93%)
Urge faecal incontinence	94 (70%)
Passive faecal incontinence	83 (61%)
Evacuatory difficulties	52 (39%)
Previous treatments for bowel dysfunction, *n* (%)	
Surgery	26 (19%)
Anterior sphincter repair	7 (5%)
Trans-vaginal rectocele repair	4 (3%)
Sacral nerve stimulation implant	4 (3%)
Laparoscopic ventral mesh rectopexy	3 (2%)
Percutaneous nerve evaluation only	3 (2%)
Injectable silicone biomaterial (PTQ^™^)	3 (2%)
Delorme’s procedure	2 (1%)
Transanal irrigation	22 (16%)
Defecating proctogram findings, *n (%)* [*n* = 60]	
Intra-rectal intussusception^±^	16 (27%)
Intra-anal intussusception^±^	25 (42%)
Rectocele^±^	38 (63%)
Enterocele^±^	2 (3%)
Endoanal ultrasound findings, *n* (%) [*n* = 112]	
Internal sphincter trauma^±^	28 (25%)
External sphincter trauma^±^	38 (34%)

*Females only

^Percentage calculated from parous females only

^+^Percentage calculated from females with vaginal deliveries only

^±^Percentage calculated from patients having undergone respective investigation

The treatment outcomes of all patients are presented in Table 2. Not every patient completed all three outcome measures. However, overall patients reported a significant reduction in median FUE (13 versus 7), UFIE (2.5 versus 1) and PFIE (2 versus 1.5; *p* < 0.05) following PTNS treatment. Similarly, patients reported a reduction in SMIS (16.5 versus 14; *p* < 0.001) and MHQ (517.5 versus 460.0; *p* < 0.001) following treatment, indicating a reported improvement in FI symptoms and a reduced impact on QoL.

**Table 2:** Overall patient-reported clinical outcome measures

Variable	Baseline	After treatment	*p*-Value
2-week bowel diary data (*n* = 110)			
Total bowel movements	34 (26–54)	33 (21–47)	0.049
Faecal urgency episodes	13 (6–21)	7 (2–18)	< 0.001
Urge faecal incontinence episodes	2.5 (0–6)	1 (0–4)	< 0.001
Passive faecal incontinence episodes	2 (0–10)	1.5 (0–6)	< 0.002
St Mark’s Incontinence Score (*n* = 126)	16.5 (13–19)	14 (10–19)	< 0.001
Manchester Health Questionnaire (*n* = 109)			
General health	25 (25–50)	25 (25–50)	0.773
Incontinence impact	75 (75–100)	75 (25–100)	< 0.001
Role impact	50 (25–62.5)	37.5 (25–56.3)	< 0.001
Physical functioning	62.5 (50–75)	50 (37.5–75)	0.004
Social functioning	50 (33.3–75)	41.7 (16.7–66.7)	< 0.001
Personal functioning	50 (0–75)	25 (0–62.5)	0.001
Emotional problems	66.7 (41.7–91.7)	50 (33.3–83.3)	< 0.001
Sleep/energy	50 (25–62.5)	37.5 (12.5–50)	0.002
Severity measures	65 (50–85)	62.5 (40–80)	< 0.001
Total	517.5 (349.2–627.5)	460 (254.6–572.1)	< 0.001

*Wilcoxon signed-rank test. Significance at the *p* < 0.05 level

When stratified according to the degree of patient-reported symptom improvement, 76 (56%) patients reported success with at least a 50% improvement in symptoms in one or more outcome measure, whilst a further 20 (15%) reported a partial response to PTNS treatment (25–50% improvement). There were 39 (29%) who reported < 25% improvement in symptoms and were considered to have failed to respond to treatment.

HRAM data was available both before and after PTNS in 121 (90%) patients. Overall, there was a reduction in the median call to stool (64 ml versus 57 ml) and maximum tolerated volume (89 ml versus 84 ml; *p* < 0.05) in addition to an increase in median ISP (27 mmHg versus 38 mmHg; *p* = 0.004; Table 3). However, when the changes in HRAM parameters are stratified according to the degree of patient-reported symptom improvement, statistically significant differences in call to stool and ISP are identified in both patients who reported treatment failure and those reporting a successful outcome (Table 4).

**Table 3:** Overall high-resolution anorectal manometry results

Measured parameter	All patients (*n* = 121)		
	Baseline	Post-PTNS	*p*-Value*
Onset (ml)	35 (25–48)	35 (23–45)	0.091
Call (ml)	64 (46–90)	57 (43–73)	**0.002**
Urge (ml)	89 (63–122)	84 (61–105)	**0.034**
MRP (mmHg)	40 (28–59)	43 (27–59)	0.969
MSP (mmHg)	77 (58–104)	85 (62–118)	0.052
ISP (mmHg)	27 (13–59)	38 (20–75)	**0.004**

Bold values indicate the significance at the *p* < 0.05 level

*Wilcoxon signed-rank test

**Table 4:** High-resolution anorectal manometry results according to symptom improvement

Measured parameter	< 25% improvement (*n* = 33)			25–50% improvement (*n* = 18)			> 50% improvement (*n* = 70)		
	Baseline	Post-PTNS	*p*-Value*	Baseline	Post-PTNS	*p*-Value*	Baseline	Post-PTNS	*p*-Value*
Onset (ml)	34 (26–50)	35 (24–47)	0.069	32 (20–48)	27 (16–39)	0.139	36 (25–46)	35 (24–48)	0.548
Call (ml)	65 (50–90)	62 (42–74)	**0.018**	44 (36–79)	47 (34–64)	0.240	65 (46–93)	58 (47–73)	**0.039**
Urge (ml)	94 (71–131)	87 (58–108)	**0.046**	64 (61–115)	74 (52–93)	0.408	96 (62–123)	85 (65–118)	0.206
MRP (mmHg)	38 (31–58)	37 (30–57)	0.262	44 (32–67)	39 (19–58)	**0.039**	40 (26–62)	49 (27–62)	0.224
MSP (mmHg)	80 (54–103)	95 (56–119)	0.331	90 (68–102)	79 (61–99)	0.492	71 (58–112)	89 (63–125)	**0.025**
ISP (mmHg)	31 (13–61)	38 (21–81)	**0.033**	29 (16–68)	39 (21–53)	0.811	26 (12–55)	36 (18–75)	**0.018**

Bold values indicate the significance at the *p* < 0.05 level

*Wilcoxon signed-rank test

Using the same thresholds of treatment success, there were no differences in baseline clinical variables, including obstetric history, symptomatology, previous treatments, FI symptom severity or endoanal ultrasound findings between patients who reported treatment failure, partial response or successful treatment. Of those who underwent defecating proctography, the finding of intra-anal intussusception was more frequently seen in patients who failed (12/19, 63%) than those who reported partial (5/11, 45%) or successful treatment (8/30, 27%; *p* = 0.040). However, there were no differences in baseline HRAM results, or in changes to HRAM parameters following PTNS between the three groups according to their reported symptom improvement (Table 5).

**Table 5:** A comparison of baseline parameters and patient-reported clinical outcomes measures according to symptom improvement

Variable	< 25% improvement (n = 39)	25–50% improvement (*n* = 20)	> 50% improvement (n = 76)	*p*-Value*
Age, median (range)	62 (32–82)	60 (34–77)	58 (27–78)	0.580
Male/female, *n* (%)	4 (10%)/35 (90%)	4 (20%)/16 (80%)	8 (11%)/68 (89%)	0.474
Obstetric history, *n* (%)				
Parous^$^	31/35 (89%)	15/16 (94%)	64/68 (94%)	0.588
Vaginal delivery^	29/35 (83%)	15/16 (94%)	64/68 (94%)	0.158
Caesarean section^	4/35 (11%)	1/16 (6%)	6/68 (9%)	0.825
Episiotomies or perineal tear^+^	24/35 (69%)	11/16 (69%)	47/68 (69%)	0.998
Bowel function history, *n* (%)				
Faecal urgency	34/39 (87%)	20/20 (100%)	72/76 (95%)	0.132
Urge faecal incontinence	27/39 (69%)	11/20 (55%)	56/76 (74%)	0.270
Passive faecal incontinence	20/39 (51%)	10/20 (50%)	53/76 (70%)	0.082
Evacuatory difficulties	14/39 (36%)	11/20 (55%)	27/76 (36%)	0.260
Previous surgery, *n* (%)	11/39 (28%)	3/20 (15%)	12/76 (16%)	0.243
Previous transanal irrigation, *n* (%)	9/39 (23%)	4/20 (20%)	9/76 (12%)	0.270
2-week bowel diary data (*n* = 110)				
Total bowel movements	29 (21–42)	41 (20–49)	34 (27–58)	0.274
Faecal urgency episodes	9 (2–24)	12 (7–17)	13 (6–22)	0.442
Urge faecal incontinence episodes	3 (0–5)	1 (0–4)	2 (0–6)	0.384
Passive faecal incontinence episodes	0 (0–4)	1 (0–12)	4 (0–11)	0.169
St Mark’s incontinence score (*n* = 126)	15 (11–18.5)	17 (14.25–20)	16 (13–19)	0.368
Manchester Health Questionnaire (*n* = 109)	500 (363.3–600)	531.7 (300–625)	510.8 (348.3–604.2)	0.789
Defecating proctogram findings (*n* = 60)				
Intra-rectal intussusception^±^	7/19 (37%)	6/11 (55%)	22/30 (73%)	**0.040**
Intra-anal intussusception^±^	12/19 (63%)	5/11 (45%)	8/30 (27%)	**0.040**
Rectocele^±^	13/19 (68%)	6/11 (55%)	19/30 (63%)	0.749
Enterocele^±^	0/19 (0%)	0/11 (0%)	2/30 (7%)	0.255
Endoanal ultrasound findings (*n* = 122)				
Internal sphincter trauma^±^	7/33 (21%)	3/16 (19%)	18/63 (29%)	0.602
External sphincter trauma^±^	11/33 (33%)	5/16 (31%)	22/63 (35%)	0.959
Baseline high-resolution anorectal manometry (*n* = 121)				
Onset (ml)	34 (26–50)	31 (20–47)	35 (24.5–46)	0.478
Call (ml)	64 (48.5–88.5)	44 (36–75)	64 (46–91.5)	0.165
Urge (ml)	84 (68.5–122)	65 (51–110)	94 (59–121.5)	0.160
MRP (mmHg)	38.2 (28.5–57.7)	43.3 (32.6–67)	40.1 (26.7–62.5)	0.967
MSP (mmHg)	77.8 (54–103.2)	93.4 (69.5–103)	71 (58.1–112.2)	0.426
ISP (mmHg)	30.9 (13.3–60.7)	30 (16.2–71.8)	26 (11.7–56.3)	0.575
Median change in high-resolution anorectal manometry parameters (*n* = 121)				
Onset (ml)	−5 (−10–3)	−6 (−18.5–5.75)	0 (−18–11)	0.513
Call (ml)	−12 (−27–5)	−4.5 (−36–9.25)	−4.5 (−28.8–12)	0.843
Urge (ml)	−6 (−33.5–10)	−3.5 (−33–21.75)	−0.5 (−29.5–16.3)	0.645
MRP (mmHg)	−1.8 (11.2–6.4)	−10.8 (−21.3–6.4)	5.4 (−10.4–16)	0.102
MSP (mmHg)	1.8 (−12.3–20.5)	−7.4 (−24.2–15.3)	10.3 (−12–34.9)	0.200
ISP (mmHg)	8.1 (−3.7–21.5)	0.2 (−10.4–17)	3.5 (−5.1–21.7)	0.606

Bold values indicate the significance at the *p* < 0.05 level

^$^Females only

^Percentage calculated from parous females only

^+^Percentage calculated from females with vaginal deliveries only

^±^Percentage calculated from patients having undergone respective investigation

*Kruskal–Wallis test or chi-squared test as appropriate

## Discussion

The mechanism of action of tibial nerve stimulation remains complex and poorly understood; however, it continues to be offered as part of the armamentarium available in the treatment of FI given it is a low-risk, minimally invasive and reversible therapy [6, 11]. This study reports a large retrospective series of patients treated with PTNS for FI in a tertiary pelvic floor referral unit. We present a holistic set of patient-reported outcome measures, including bowel diary data, symptom severity and QoL questionnaire results capturing various aspects of the lived experience of FI alongside high-resolution anorectal manometry results. It demonstrates that some patients report PTNS improves symptoms of FI. In our series, up to 56% reported at least a 50% improvement in symptoms in the short-term at 6 to 12 weeks follow-up. This ‘success’ rate is lower than that reported in a systematic review of PTNS efficacy (62–83%) using the same definition of success (≥ 50% improvement) [6], although our study is larger than any of the included series (range: 10–88). This arbitrary ‘50% improvement’ definition of ‘success’ is ubiquitous in studies of neuromodulation in FI, although work is now underway to develop and agree on FI specific outcome measures [17, 18]. However, recognising patients suffering with FI may be satisfied with a modest improvement in symptoms of 25%, up to 71% of patients in our series reported benefit using this threshold. Our results suggest that patients -report PTNS may be most effective in reducing episodes of faecal urgency and urge faecal incontinence, a finding that has previously been demonstrated in the CONFIDeNT trial [9]. Indeed, urgency represents the main indication for PTNS therapy in urinary dysfunction [19]. Despite these patient-reported benefits, the clinical outcomes of PTNS have been suggested to be a result of the placebo effect of regular interaction with a continence therapist [20], or in the natural change in FI over time that is responding to other non-surgical treatment including lifestyle modifications and pharmacological therapy [21]. The CONFIDeNT trial [9], and other randomised controlled studies of PTNS [22] and sacral neuromodulation [23], have reported clinical improvement in all their sham treatment arms, indicating that a placebo effect contributes to the overall patient-reported benefit. In our study, the patient-reported benefits with PTNS were independent to the physiological changes measured with HRAM. Whilst there were statistically significant reductions in rectal sensory volumes and an increase in anal canal sphincter pressures, these changes were seen in both groups reporting symptom improvement and those who did not. In addition, the baseline HRAM results, or the changes following treatment, did not correspond with the degree of symptom improvement reported by patients. When considering pre-treatment variables, only the presence of intra-anal rectal intussusception appeared to be more prevalent in those who reported a failure of PTNS (< 25% symptom improvement).

PTNS was first proposed as a treatment for FI in 2003 [24] based on the hypothesis that stimulation of the posterior tibial nerve could result in similar effects seen with SNM given the shared origin with the pudendal nerve in the sacral nerve roots [8]. Whilst the exact mechanism of action remains elusive, several authors have attempted to measure the physiological effects of tibial nerve stimulation using anorectal manometry. Various results have been presented, including no changes in resting or squeeze pressure [13, 25–27], an improvement in MSP alone [28] or an improvement in both MRP and MSP [29, 30]. In one randomised study, the authors noted the same changes in sphincter pressures in both the active and sham treatment arms [22]. In two randomised studies, rectal sensory volumes were unchanged following tibial nerve stimulation [22, 26] whilst demonstrating a non-significant reduction in another series [27]. When considering baseline measurements, none of these parameters have demonstrated an ability to predict the success of PTNS treatment [31], a finding that is supported by the results in our series. These mixed results, in the context of improvements in patient-reported symptoms, may either reflect that what is being measured by anorectal manometry might not be what is relevant to understand the mechanistic pathways, or that the placebo effect of PTNS is significantly contributing to the reported benefit, or both. Similar conclusions have also been described in studies of SNM mechanisms [32]. In our series, we identified a statistically significant reduction in rectal sensory thresholds and an increase in ISP; however, these findings were also identified in patients who reported < 25% improvement in their symptoms. In addition, whilst the results are statistically significant, it is difficult to reconcile that these clinically small changes in HRAM parameters could lead to a significant improvement in FI symptoms reported by the patients. It is also recognised that a key limitation in our retrospective data is that HRAM performed before PTNS treatment was often undertaken during trials of other conservative interventions, a factor that cannot be controlled for in this retrospective study, or any others to our knowledge. The reasons underlying the mismatch between the bowel diary and questionnaire improvements and HRAM findings in our series and others may lie in that the principal effect of PTNS has been suggested to be on afferent nerve function, which cannot be measured with HRAM [33]. This may help to explain the efficacy of PTNS in the treatment of faecal urgency and why changes in the anorectum measured with conventional tests fail to correspond with the symptom improvement reported by patients [33, 34]. This should remain an area for future research with other novel tests of the continence mechanism [35].

The result of the randomised CONFIDeNT trial [9], which failed to demonstrate a significant difference between PTNS and sham, was later questioned, as with the exclusion of patients with concomitant obstructed defecation symptoms, a significant clinical effect of PTNS was demonstrated. The presence of patient-reported evacuatory difficulties in our series was no different between the groups of patients stratified according to treatment response. However, in the 60 patients who underwent defecating proctography before PTNS as part of their routine evaluations, there was a greater incidence of high grade intra-anal rectal intussusception amongst patients who reported < 25% improvement in symptoms (*p* = 0.040). The negative effects of rectal intussusception on neuromodulation outcomes have been highlighted previously [10, 36], and it has been suggested that correction of these anatomical abnormalities first may improve the efficacy of neuromodulation treatments [37]. Whether therefore the symptoms of obstructed defecation, or the presence of high-grade intra-anal rectal intussusception, should feature as an exclusion criterion for PTNS remains uncertain and warrants further consideration.

This single-centre series represents a large cohort of patients treated with PTNS for FI in ‘real-world’ clinical practice and demonstrates patients report it offers some benefit in ameliorating FI symptoms, which is independent of HRAM results. This may either reflect that HRAM is unable to measure the clinical effect of PTNS, or that the principal reason for the reported benefit is a placebo effect, or both. There are however several limitations with this study which impact the findings. The retrospective design with missing outcome data in some patients may introduce selection bias, and its single-centre nature limits the external validity of the findings, whilst not all patients underwent the same investigations before starting PTNS treatment. In addition, it is not possible to quantify the ongoing effect of other non-surgical interventions such as lifestyle modifications, diet and medications that may be contributing to the reported efficacy of PTNS, particularly where the reported benefit was small. The results presented here only represent short-term follow-up (6–12 weeks), leaving the long-term outcomes of these patients unclear. This makes counselling patients, and understanding the impact of the placebo effect, more challenging, as it has not been established whether the reported clinical benefit is sustained after cessation of the treatment. Finally, the treatment course used here was of 12 weekly consecutive treatments and was completed by most patients. However, other authors have advocated different treatment regimens [38] with ‘top-up’ therapies or prolonged courses of up to 12 months to maintain, or improve efficacy [39, 40]. However, whilst patients may value regular hospital visits to receive PTNS therapy delivered by a continence therapist, others may either be unable to commit to these visits over 12 weeks or are unable to travel at all to a unit delivering this therapy. Home-based PTNS has been suggested as a safe and feasible option to improve compliance with treatment in highly selected patients [41], whilst dorsal genital nerve simulation has also been proposed as an alternative therapy delivered by patients at home [42]. Further work is now required to establish the role and mechanism of action of PTNS therapy in FI, and to specifically identify those patients in whom PTNS is likely to be efficacious, a conclusion acknowledged by other authors [9, 10].

## Conclusion

Patients report PTNS therapy may offer benefit in ameliorating FI symptoms, particularly with faecal urgency. In the short-term, 56% of patients reported at least a 50% improvement in their symptoms following 12 weeks of treatment. Despite this reported clinical improvement, changes in HRAM parameters were no different between patients who reported successful treatment and those who did not, raising the possibility of, at least in part, a placebo effect contributing to the findings. Only the presence of intra-anal rectal intussusception identified on defecating proctography was significantly more common in patients who reported < 25% improvement in symptoms (*p* = 0.040) indicating this finding may predict a suboptimal PTNS result. Further work is therefore required to establish the efficacy of PTNS in the context of a plausible placebo effect and its mechanistic pathways and identify subgroups who will most likely benefit.

## Data Availability

All data described in this manuscript have been presented in the relevant figures and tables. Data can be made available upon reasonable request to the corresponding author.
